# Investigating the Correlation–Firing Rate Relationship in Heterogeneous Recurrent Networks

**DOI:** 10.1186/s13408-018-0063-y

**Published:** 2018-06-06

**Authors:** Andrea K. Barreiro, Cheng Ly

**Affiliations:** 1 Department of Mathematics, Southern Methodist University, Dallas, USA; 2 Department of Statistical Science and Operations Research, Virginia Commonwealth University, Richmond, USA

**Keywords:** Recurrent network, Spike count correlation, Linear response, Heterogeneity

## Abstract

The structure of spiking activity in cortical networks has important implications for how the brain ultimately codes sensory signals. However, our understanding of how network and intrinsic cellular mechanisms affect spiking is still incomplete. In particular, whether cell pairs in a neural network show a positive (or no) relationship between pairwise spike count correlation and average firing rate is generally unknown. This relationship is important because it has been observed experimentally in some sensory systems, and it can enhance information in a common population code. Here we extend our prior work in developing mathematical tools to succinctly characterize the correlation and firing rate relationship in heterogeneous coupled networks. We find that very modest changes in how heterogeneous networks occupy parameter space can dramatically alter the correlation–firing rate relationship.

## Introduction

One prominent goal of theoretical neuroscience is to understand how spiking statistics of cortical networks are modulated by network attributes [9, 28, 42]. This understanding is essential to the larger question of how sensory information is encoded and transmitted, because the statistics of neural activity impact population coding [7, 15–17, 37]. One family of statistics that is implicated in nearly all population coding studies is trial-to-trial variability (and co-variability) in spike counts; there is now a rich history of studying how these statistics arise, and how they effect coding of stimuli [1, 10, 18, 25, 33]. Recent work has demonstrated that the information content of spiking neural activity depends on spike count correlations and its relationship (if any) with stimulus tuning [1, 6, 18, 25, 44].

An important relationship observed in many experimental studies is that pairwise correlations on average increase with firing rates. This has been observed *in vitro* [8] and in several visual areas: area MT [2], V4 [5] (when measured between cells in the same attentional state), V1 [19, 36], and notably, in ON–OFF directionally sensitive retinal ganglion cells [11, 44]. The retinal studies involved cells with a clearly identified function, and therefore allowed investigation of the coding consequences of the observed correlation–firing rate relationship. These studies found that the *stimulus-dependent* correlation structure observed compared favorably to a structure in which *stimulus-independent* correlations were matched to their (stimulus-)averaged levels. This finding reflects a general principle articulated in other studies [18, 25], that stimulus-dependent correlations are beneficial when they serve to spread the neural response in a direction *orthogonal* to the signal space.

These findings thus provide strong motivation for understanding what network mechanisms can produce this positive (and perhaps beneficial) correlation–firing rate relationship. This correlation–firing rate trend has been explained theoretically in feedforward networks driven by common input [8, 26, 38]; however, many cortical networks are known to be dominated by strong recurrent activity [24, 34, 39]. On the other hand, theoretical studies of the mechanisms for correlations in recurrent networks have largely analyzed homogeneous networks (i.e., identical cells, aside from excitatory/inhibitory cell identity) [9, 13, 27, 28, 40, 41], and have not considered how correlations vary with firing rates with realistic intrinsic heterogeneity. Thus, how spike count correlations can vary across a population of heterogeneously-tuned, recurrently connected neurons is not yet well understood despite its possible implications for coding.

In a previous paper, we addressed this gap by developing a mathematical method to compactly describe how second-order spike count statistics vary with both intrinsic and network attributes [4]. Specifically, we adapted network linear response theory [14, 27, 43] to account for heterogeneous and recurrent networks, which in turn allows us to identify important network connections that contribute to correlations via a single-cell susceptibility function [8]. Here, we will use this method to survey a broad family of recurrent networks to understand how three factors influence the relationship between correlations and firing rates; how the neurons occupy parameter space, the strength of recurrent excitation, and the strength of background noise. This work thus provides a more complete picture of how even modest changes in important circuit parameters can alter the correlation–firing rate relationship.

## Results

First, we summarize how a network linear response theory allows us to use the single-neuron firing rate function to approximate correlations. We then apply this theory to examine three factors that can modulate the correlation–firing rate relationship: direction in effective parameter space, strength of recurrent excitation, and strength of background noise.

### Using the Single-Neuron Firing Rate Function to Characterize Correlation Susceptibility

The response of a neuron is generally a nonlinear function of model parameters. However, background noise linearizes this response, so that we can use a linear theory to describe the change in response to small changes in parameters. We assume we have some way to approximate the change in firing rate which occurs as a result of a change in parameter *X*:

1 $$\begin{aligned} \nu_{i} (t) = & \nu_{i,0} + (A_{X,i} * X_{i}) (t); \end{aligned}$$

$\nu_{i,0}$ is the baseline rate (when $X = 0$) and $A_{X,i}(t)$ is a *susceptibility function* that characterizes the firing rate response [8, 20, 41]. The parameter which is modulated has often been chosen to be a current bias *μ* [8, 41]; however, it could also be the mean or variance of a conductance, a time constant, or a spike generation parameter [29, 30].

In a coupled network, the parameter change $X_{i}$ arises from inter-neuron coupling; substituting $X_{i}(t) \rightarrow \sum_{j} (\mathbf {J}_{X,ij} * \nu_{j} )$ and moving to the spectral domain, we find

2 $$\begin{aligned} \bigl\langle \tilde{y}(\omega) \tilde{y}^{\ast}(\omega) \bigr\rangle = & \bigl( \mathbf {I}- \tilde{\mathbf {K}}(\omega) \bigr)^{-1} \bigl\langle \tilde {y}^{0}(\omega) \tilde{y}^{0 \ast}(\omega) \bigr\rangle \bigl( \mathbf {I}- \tilde{\mathbf {K}}^{\ast}(\omega) \bigr)^{-1}, \end{aligned}$$

where $\tilde{y}_{i} = \mathcal{F} [ y_{i} - \nu_{i} ]$ is the Fourier transform of the mean-shifted process ($\nu_{i}$ is the average firing rate of cell *i*) and $\tilde{f} = \mathcal{F} [ f ]$ for all other quantities; $\tilde{\mathbf {K}}_{ij} (\omega) = \tilde {A}_{X, i}(\omega) \tilde{\mathbf {J}}_{X, ij}(\omega)$ is the interaction matrix in the frequency domain (which may depend on the parameter being varied, i.e. *X*); $\langle\tilde{y}^{0}(\omega) \tilde{y}^{0 \ast}(\omega) \rangle$ is the power spectrum of the uncoupled neural response. Using the usual series expansion for $( \mathbf {I}- \tilde{\mathbf {K}}(\omega) )^{-1}$, we see that the covariance $\tilde{\mathbf {C}} (\omega) \equiv\langle\tilde{y}(\omega) \tilde{y}^{\ast}(\omega ) \rangle$ naturally decomposes into contributions from different graph motifs:

3 $$\begin{aligned} \tilde{\mathbf {C}} (\omega) =& \bigl( \mathbf {I}- \tilde{\mathbf {K}}(\omega) \bigr)^{-1} \tilde{\mathbf {C}}^{0} (\omega) \bigl( \mathbf {I}- \tilde{ \mathbf {K}}^{\ast}(\omega) \bigr)^{-1} \\ = & \tilde{\mathbf {C}}^{0} (\omega) + \tilde{\mathbf {K}}(\omega) \tilde { \mathbf {C}}^{0} (\omega) + \tilde{\mathbf {C}}^{0} (\omega) \tilde{\mathbf {K}}^{*}(\omega) + \tilde{\mathbf {K}}(\omega) \tilde{\mathbf {C}}^{0} (\omega) \tilde{ \mathbf {K}}^{*}(\omega) + \cdots . \end{aligned}$$

Each instance of $\tilde{\mathbf {K}}$ includes the susceptibility function in the spectral domain, $A_{X}(\omega)$, which modulates the effect of each directed connection by the responsiveness of the target neuron to the parameter of interest. As noted by previous authors [27], the validity of the expansion in Eq. (3) relies on the spectral radius of *K̃*, $\rho(\tilde{K})$, remaining less than one. We checked that this remains true for all networks we examined in this paper, with a maximum of $\rho(\tilde{K}) = 0.9564$.

We next justify why—at least for long-time correlations—we can estimate each of these terms using the single-neuron firing rate function. First, in the small frequency limit, $A_{X,i}(\omega)$ will coincide with the derivative of the firing rate with respect to the parameter:

$$\lim_{\omega\rightarrow0} \tilde{A}_{X,i}(\omega) = \frac{d\nu _{i}}{d X}. $$

For the common input motif, $\tilde{\mathbf {K}}(\omega) \tilde{\mathbf {C}}^{0} (\omega) \tilde{\mathbf {K}}^{*}(\omega)$, we can write

4 $$\begin{aligned} \bigl( \tilde{\mathbf {K}} \tilde{\mathbf {C}}^{0} \tilde{\mathbf {K}}^{*} \bigr)_{ij} = &\sum_{k \rightarrow i, k\rightarrow j} \tilde{\mathbf {K}}_{ik} \tilde{\mathbf {C}}^{0}_{kk} \tilde{ \mathbf {K}}_{jk} \end{aligned}$$

5 $$\begin{aligned} = &\sum_{k \rightarrow(i,j), k \in E} (\tilde{A}_{g_{E},i} \tilde{ \mathbf {J}}_{ik} ) \tilde{\mathbf {C}}^{0}_{kk} (\tilde {A}_{g_{E},j} \tilde{\mathbf {J}}_{jk} ) \\ &{} + \sum _{k \rightarrow(i,j), k \in I} (\tilde{A}_{g_{I},i} \tilde{\mathbf {J}}_{ik} ) \tilde{\mathbf {C}}^{0}_{kk} (\tilde{A}_{g_{I},j} \tilde{ \mathbf {J}}_{jk} ) \end{aligned}$$

6 $$\begin{aligned} = & \vert \tilde{\mathbf {J}}_{E} \vert ^{2} \sum _{k \rightarrow(i,j), k \in E} \tilde{A}_{g_{E},i} \tilde{A}_{g_{E},j} \tilde{\mathbf {C}}^{0}_{kk} \\ &{} + \vert \tilde{\mathbf {J}}_{I} \vert ^{2} \sum_{k \rightarrow(i,j), k \in I} \tilde{A}_{g_{I},i} \tilde {A}_{g_{I},j} \tilde{\mathbf {C}}^{0}_{kk} \end{aligned}$$

7 $$\begin{aligned} = & \vert \tilde{\mathbf {J}}_{E} \vert ^{2} \tilde{A}_{g_{E},i} \tilde{A}_{g_{E},j} \sum _{k \rightarrow(i,j), k \in E} \tilde {\mathbf {C}}^{0}_{kk} \\ &{} + \vert \tilde{\mathbf {J}}_{I} \vert ^{2} \tilde {A}_{g_{I},i} \tilde{A}_{g_{I},j} \sum_{k \rightarrow(i,j), k \in I} \tilde{ \mathbf {C}}^{0}_{kk}, \end{aligned}$$

where we have separated excitatory (E) and inhibitory (I) contributions, using $g_{E}$ and $g_{I}$ to denote the mean conductance of each type, and assumed that the synaptic coupling kernels, $\tilde {\mathbf {J}}_{jk}$, depend only on E/I cell identity (and have thus dropped the first subscript, which adds no additional information).

This provides a formula for the long-time *covariance*, but we are typically interested in the *correlation*; fortunately, the small frequency limit also allows us to obtain a normalized correlation measure from the cross-spectrum, because

8 $$\begin{aligned} \lim_{T \rightarrow\infty} \rho_{T,ij} = & \lim_{T \rightarrow \infty} \frac{\operatorname {Cov}_{T}(n_{i},n_{j})}{\sqrt{ \operatorname {Var}_{T}(n_{i}) \operatorname {Var}_{T}(n_{j})}} = \frac{\tilde{\mathbf {C}}_{ij}(0)}{\sqrt{\tilde{\mathbf {C}}_{ii}(0) \tilde {\mathbf {C}}_{jj}(0)}}, \end{aligned}$$

where $\operatorname {Cov}_{T}(n_{i},n_{j})$ and $\operatorname {Var}_{T}(n_{i})$ denote covariance and variance of spike counts in a time window *T*; that is, $\rho_{T,ij}$ is the Pearson correlation coefficient (which varies between −1 and 1).

Finally, letting $\omega\rightarrow0$ and normalizing with the assumption that spiking is close to a Poisson process, as expected for an asynchronously firing network, so that $\tilde{\mathbf {C}}_{kk} \approx \nu_{k}$:

9 $$\begin{aligned} \frac{ ( \tilde{\mathbf {K}} \tilde{\mathbf {C}}^{0} \tilde{\mathbf {K}}^{*} )_{ij}}{\sqrt{\tilde{\mathbf {C}}_{ii} \tilde{\mathbf {C}}_{jj} }} = & \underbrace{ \frac{1}{\sqrt{\nu_{i} \nu_{j}}} \frac{d\nu_{i}}{d g_{E}} \frac{d\nu_{j}}{d g_{E}} }_{\substack{\text{modulation by}\\ \text{firing rate function}}} \vert \tilde{\mathbf {J}}_{E} \vert ^{2} \underbrace{ \biggl[ \sum_{\substack{k \rightarrow(i,j), \\ k \in E}} \tilde{\mathbf {C}}^{0}_{kk} \biggr]}_{\text{total E common input}} \\ &{} + \underbrace{ \frac{1}{\sqrt{\nu_{i} \nu_{j}}} \frac{d\nu_{i}}{d g_{I}} \frac{d\nu_{j}}{d g_{I}} }_{\substack{\text{modulation by}\\ \text{firing rate function}}} \vert \tilde{\mathbf {J}}_{I} \vert ^{2} \underbrace{ \biggl[ \sum _{ \substack{k \rightarrow(i,j),\\ k \in I} } \tilde{\mathbf {C}}^{0}_{kk} \biggr]}_{\text{total I common input}} . \end{aligned}$$

The above expression summarizes how the impact of excitatory and inhibitory common input are modulated by the single-neuron firing rate function, *ν*, and its derivatives.

Thus far, we have presented results previously obtained by others [27, 40, 41]. We now depart from these authors by focusing specifically on the behavior of the term in Eq. (9); and for simplicity, the behavior of this modulating factor for two identical neurons; e.g.

10 $$\begin{aligned} \frac{1}{\sqrt{\nu_{i} \nu_{j}}} \frac{d\nu_{i}}{d g_{I}} \frac{d\nu _{j}}{d g_{I}} \rightarrow& \frac{1}{\nu} \biggl( \frac{d\nu}{d g_{I}} \biggr)^{2}. \end{aligned}$$

In principle, analogous expressions govern larger motifs, such as chains, that involve additional evaluations of *ν* and its derivatives; for example, excitatory length-3 chains arising from $\tilde{\mathbf {K}}^{3} \tilde{\mathbf {C}}^{0}$ would be:

11 $$\begin{aligned} \frac{ ( \tilde{\mathbf {K}}^{3} \tilde{\mathbf {C}}^{0} )_{ij}}{\sqrt {\tilde{\mathbf {C}}_{ii} \tilde{\mathbf {C}}_{jj} }} = & \underbrace{ \biggl[ \frac{1}{\sqrt{\nu_{i} \nu_{j}}} \frac{d\nu_{i}}{d g_{E}} \frac{d\nu_{l}}{d g_{E}} \frac{d\nu_{k}}{d g_{E}} \biggr]}_{\text{modulation by firing rate function}} \times \vert \tilde{\mathbf {J}}_{E} \vert ^{3} \times \underbrace{ \biggl[ \sum_{l \rightarrow i} \sum _{\substack{k \rightarrow l,\\ l \in E}} \sum_{\substack{j \rightarrow k,\\ k \in E}} \tilde{ \mathbf {C}}^{0}_{jj} \biggr]}_{\text{all $E \rightarrow E \rightarrow E \rightarrow E$ paths}} . \end{aligned}$$

However, we found that, for a wide range of networks, direct common input—and *inhibitory* common input in particular—was the dominant contributor to pairwise correlations (Fig. 6(A)).

We now examine how different network mechanisms modulate the correlation–firing rate relationship, focusing on three factors: direction in effective parameter space, strength of recurrent excitation, and strength of background noise.

### Direction in Parameter Space Modulates the Correlation–Firing Rate Relationship

Suppose we have a firing rate function of one or more intrinsic parameters (for exposition purposes, assume a function of two parameters $(x_{1}, x_{2})$), i.e.

$$\nu= F(x_{1}, x_{2}). $$

The parameters $x_{j}$ might include input bias, membrane time constant, ionic/synaptic reversal potentials, or a spiking threshold. Based on our arguments from the previous section, we will approximate *correlation susceptibility* by the quantity

$$\hat{S} = \frac{1}{F} \biggl( \frac{\partial F}{\partial x_{1}} \biggr)^{2}, $$

where $x_{1}$ is an appropriately chosen parameter. Specifically, we will find, empirically, that the inhibitory common input is the dominant term, and therefore will use $x_{1} = g_{I}$, the mean inhibitory conductance. Thus, the units of *Ŝ* in all figures are Hz/[$g_{I}^{2}$], where $g_{I}$ is dimensionless (see Eq. (17)).

Heterogeneous firing rates can arise when each neuron occupies a different location in parameter space (i.e. a different $(x_{1},x_{2})$ point), thus causing both firing rate *F* and susceptibility *Ŝ* to vary from neuron to neuron. We now ask: how does *Ŝ* change with firing rate?

Note that this question, as stated, is ill-posed because *F* and *Ŝ* are both functions of two parameters ($x_{1}$ and $x_{2}$): there is not necessarily a one-to-one or even a functional relationship between these quantities. Suppose that, locally, a population of neurons can be described as lying along a parameterized path in the $(x_{1}, x_{2})$ plane: i.e., $(x_{1}(s), x_{2}(s))$, for $s_{\mathrm{min}}< s< s_{\mathrm{max}}$. Then we can compute the directional derivative:

12 $$ \frac{d\hat{S}}{dF} = \frac{d\hat{S}/ds}{ dF/ds} = \frac{\nabla\hat{S} \cdot d\mathbf {x}}{ \nabla F \cdot d\mathbf {x}}, $$

where we have expressed the directional derivatives in terms of the local direction of the path: i.e. $d \mathbf {x}= (\frac{dx_{1}}{ds}, \frac {dx_{2}}{ds})$ and the gradients of *F* and *Ŝ*. However, this depends not only on the functions *F* and *Ŝ*, but also on the direction *d***x**.

**Fig. 1:**
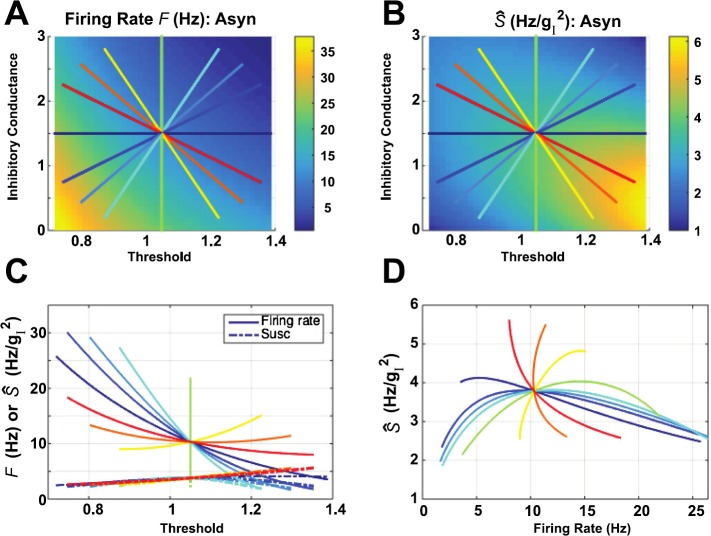
Firing rate and susceptibility (*Ŝ*), computed for the asynchronous (Asyn) network studied in [4], with all other parameters besides threshold *θ* and mean inhibitory conductance $\langle g_{I}\rangle$ set to their average values (thereby leaving a two-dimensional parameter space). Here, we traverse the plane on straight-line paths defined by their angle through the origin. Although the units of $g_{I}$ are dimensionless, they are shown as the units for *Ŝ* for completeness. The units of *θ* (i.e., voltage) are also scaled to be dimensionless

To gain some intuition for why (and when) direction in $(x_{1},x_{2})$ space matters, we consider the networks studied in [4]. Previously, we simplified the firing rate function by setting all but two parameters (inhibitory conductance, $\langle g_{I,i} \rangle$, and threshold, $\theta_{i}$) to their population average; i.e.

13 $$\begin{aligned} F \bigl( \langle g_{I,i} \rangle, \theta_{i} \bigr) \equiv& f \bigl( \langle g_{I,i} \rangle, \langle\sigma_{g_{I},i} \rangle_{p}, \bigl\langle \langle g_{E,i} \rangle \bigr\rangle _{p}, \langle\sigma_{g_{E},i} \rangle _{p}, \langle\sigma_{i} \rangle_{p}, \theta_{i} \bigr) , \end{aligned}$$

and $\langle \cdot \rangle_{p}$ denotes the population average. In Figs. 1(A) and (B), we show *F* and $\hat{S} \equiv ( \frac{\partial F}{\partial x_{1}} )^{2} /F$ thus computed, for the asynchronous network studied in that paper. We then surveyed a sequence of diagonal paths through the center (i.e., midpoint of the ranges of threshold and inhibitory conductance), with each path plotted in a different color. In Fig. 1(C) we plot firing rate (solid lines) and susceptibility (dashed-dotted lines) along each curve. Finally, in Fig. 1(D) we plot the susceptibility versus the firing rate, along each path.

This last panel shows that there is striking variability in the susceptibility-firing rate relationship, depending on the direction the path takes through the center of the $(\theta, \langle g_{I} \rangle )$ plane. Any given firing rate (below $\sim15\mbox{ Hz}$) is consistent with either increase or decrease of susceptibility. All curves go through a single point in the $(\theta, \langle g_{I} \rangle)$ plane, and therefore a single point in the $(F, \hat{S})$ plane; here the direction of the *Ŝ*–*F* relationship (i.e., whether *Ŝ* increases or decreases with *F*) can change rapidly with angle, as for the red, orange, and yellow curves.

**Fig. 2:**
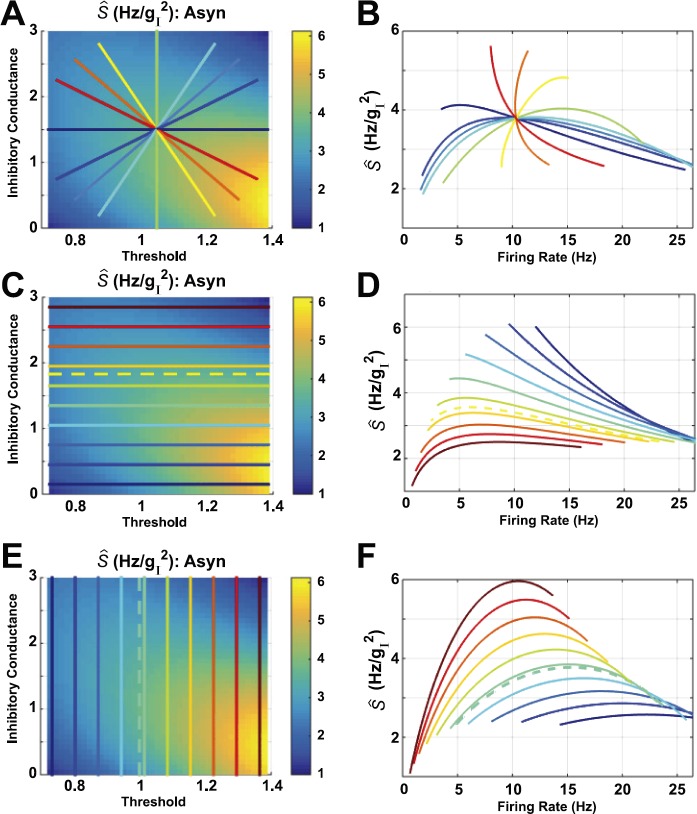
Susceptibility (*Ŝ*) vs. firing rate, computed for the asynchronous network studied in [4], with all other parameters besides threshold *θ* and mean inhibitory conductance $\langle g_{I}\rangle$ set to their average values (thereby leaving a two-dimensional parameter space: the other (averaged) parameters are $\langle\sigma_{g_{I},i} \rangle_{p} = 0.6602$ (see Eq. (30)), $\langle\sigma_{g_{E},i} \rangle_{p} = 0.0026$ (see Eq. (29)), $\langle\sigma_{i} \rangle_{p} = 6.3246$, $\langle\langle g_{E},i \rangle \rangle_{p} = 0.0053$ (see Eq. (17)). Here, we traverse the plane on three different families of straight-line paths. The dashed lines show paths visualized in [4]: $\theta= 1$ (vertical, aqua blue) and $\langle g_{I}\rangle= 1.83$ (horizontal, orange/yellow)

We then extended these observations by traversing the phase space with two additional families of straight-line paths (Fig. 2); the radial paths are repeated in Figs. 2(A) and (B). For horizontal (Figs. 2(C) and (D)) and vertical (Figs. 2(E) and (F)) families, paths no longer intersect at a single point; nevertheless, a given firing rate is consistent with both increases and decreases in susceptibility. This is pronounced in Fig. 2(F), where at 15 Hz susceptibility decreases with firing rate in the orange, yellow and light green paths, but increases for the light blue, medium blue, royal blue, and indigo paths.

**Fig. 3:**
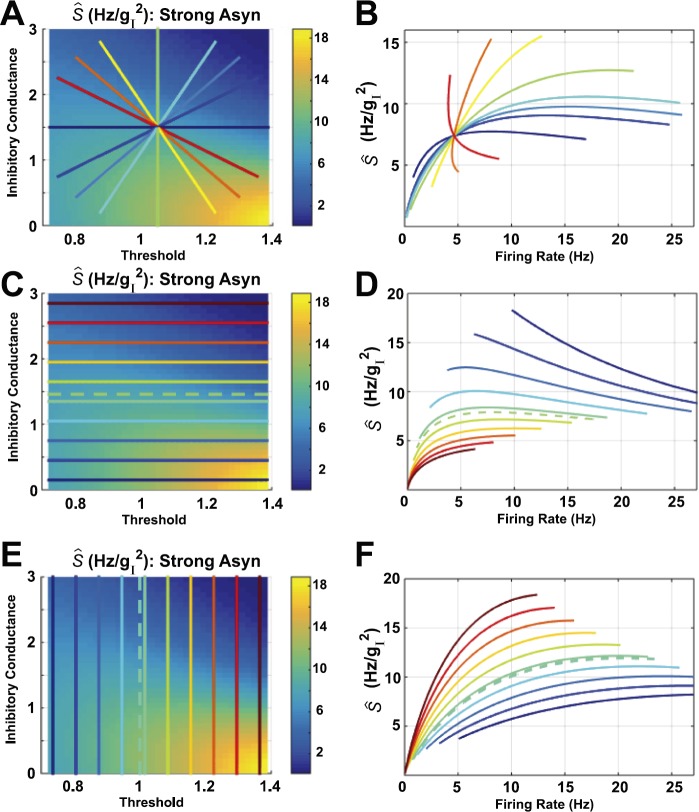
Susceptibility (*Ŝ*) vs. firing rate, computed for the strong asynchronous (Strong Asyn) network studied in [4], with all other parameters besides threshold *θ* and mean inhibitory conductance $\langle g_{I}\rangle$ set to their average values (thereby leaving a two-dimensional parameter space: the other (averaged) parameters are $\langle\sigma_{g_{I},i} \rangle_{p} = 0.5884$ (see Eq. (30)), $\langle\sigma_{g_{E},i} \rangle_{p} = 0.0378$ (see Eq. (29)), $\langle\sigma_{i} \rangle_{p} = 4.7434$, $\langle \langle g_{E},i \rangle \rangle_{p} = 0.0611$, see Eq. (17)). Here, we traverse the plane on three different families of straight-line paths. Dashed lines show paths visualized in [4]: $\theta= 1$ (vertical, aqua blue) and $\langle g_{I}\rangle= 1.46$ (horizontal, yellow/green)

We performed the same computations on the *strong asynchronous* network studied in [4] that has larger excitatory coupling strength: results are shown in Fig. 3. A given firing rate could be consistent with either increase or decrease of susceptibility; we see this in the radial paths (Figs. 3(A) and (B)) and horizontal paths (Figs. 3(C) and (D)) for rates between 5–10 Hz. However, vertical paths (Figs. 3(E) and (F)) always have susceptibility increasing with firing rate (except perhaps at the highest firing rates). As in the asynchronous network, direction of susceptibility (increase vs. decrease) can change rapidly with angle, for paths that intersect a single point; see Figs. 3(A)–(B), red to orange to yellow.

### Quantifying the Likelihood of a Positive Correlation–Firing Rate Relationship

**Fig. 4:**
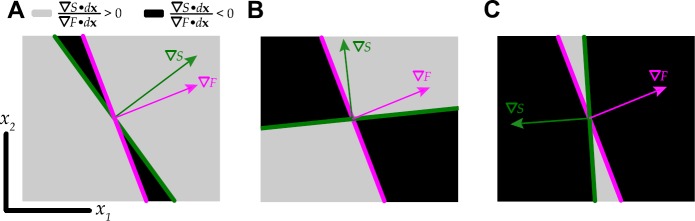
Where ∇*Ŝ* and ∇*F* are similarly aligned, *Ŝ* and *F* will both increase along most paths through that point. In each panel, gray shows the part of the **x**-plane where $\frac{d\hat{S}}{dF} = \frac{\nabla\hat{S} \cdot d\mathbf {x}}{\nabla F \cdot d\mathbf {x}} > 0$, black where $\frac{d\hat{S}}{dF}<0$. From left to right: ∇*Ŝ* and ∇*F* positively aligned; ∇*Ŝ* and ∇*F* orthogonal; ∇*Ŝ* and ∇*F* negatively aligned

In the previous section, we saw that the path a network occupies in effective parameter space can have a dramatic effect on the correlation–firing rate relationship: we next seek to formalize these observations. Let *d***x** be the local direction in which we want to consider the behavior of *F* and *Ŝ*. If $\nabla\hat{S} \cdot d \mathbf {x}$ and $\nabla F \cdot d \mathbf {x}$ are of the same sign, then *Ŝ* increases with *F*; if $\nabla\hat {S} \cdot d \mathbf {x}$ and $\nabla F \cdot d \mathbf {x}$ have opposite signs, then *Ŝ* decreases with *F*. The more aligned ∇*Ŝ* and ∇*F*, the more paths lead to $\frac{d\hat{S}}{dF} > 0$; the more anti-aligned ∇*Ŝ* and ∇*F*, the more paths lead to $\frac{d\hat{S}}{dF} < 0$. For example, consider the limiting cases where: (i) if ∇*Ŝ* and ∇*F* point exactly in the same direction, then $\nabla\hat {S} \cdot d \mathbf {x}$ and $\nabla F \cdot d \mathbf {x}$ are always same-signed for any *d***x**; (ii) if ∇*Ŝ* and ∇*F* point in opposite directions, then $\nabla\hat{S} \cdot d \mathbf {x}$ and $\nabla F \cdot d \mathbf {x}$ are never same-signed. Figure 4 illustrates how the alignment of these two quantities alters the region where correlation increases with firing rate.

**Fig. 5:**
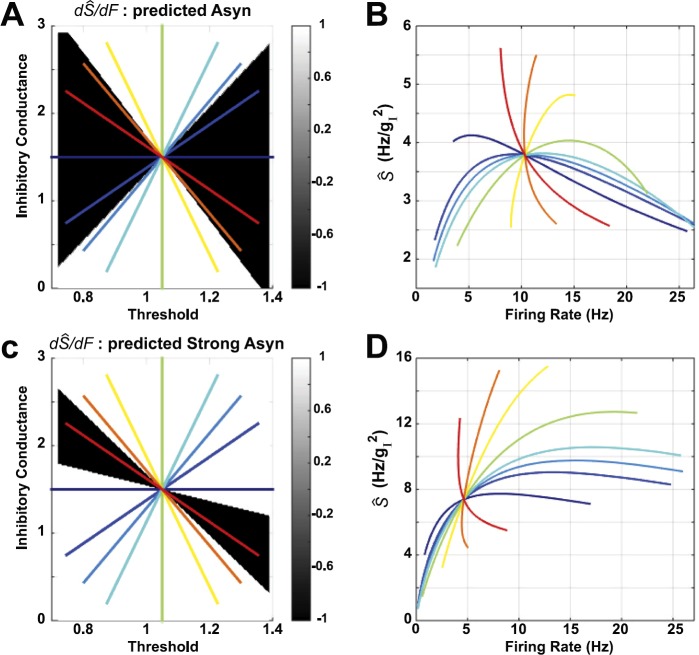
Using a single number to predict $d\hat{S}/dF$. (A) Paths through parameter space for the asynchronous network: white shows the part of the **x**-plane where $\frac{d\hat{S}}{dF} = \frac{\nabla \hat{S} \cdot d\mathbf {x}}{\nabla F \cdot d\mathbf {x}} > 0$, black where $\frac{d\hat{S}}{dF}<0$, where ∇*Ŝ* and ∇*F* are computed at the center of the diagram. (B) Correlation susceptibility vs. firing rate, for each path illustrated in (A). (C)–(D) As in (A)–(B), but for the strong asynchronous network

To examine the utility of this idea, we reconsider the radial paths along which we previously computed susceptibility. The paths all go through a single point, so we can check the $\operatorname{sign} ( (\nabla\hat{S} \cdot \mathbf {x}) (\nabla F \cdot \mathbf {x}) )$ for all directions through this point. In Figs. 5(A) and (C), white indicates positive and black negative. Figures 5(B) and (D) repeat the susceptibility-firing rate curves from Fig. 2(B) and Fig. 3(B). For the asynchronous network (Fig. 5(A)), the red, indigo, and royal blue paths are predicted to have negative $d\hat {S}/dF$, as we can confirm in Fig. 5(B). Yellow, green, and light blue curves are predicted to have positive $d\hat{S}/dF$. The orange curve is close to $dF = 0$; the true blue curve is close to $dS = 0$. For the strong asynchronous network, only the red curve is in the negative region of Fig. 5(C); this is also the only path with $d\hat {S}/dF < 0$ in Fig. 5(D).

Of course, this prediction only applies to portions of the path near the point at which we computed the gradients; away from this point, gradients will change along with the direction of the *Ŝ* vs. *F* curve. For example, the royal blue curve in Fig. 5(B) increases with firing rate for small firing rates, and the light blue, true blue, and royal blue curves in Fig. 5(D) decrease with firing rate, (for large firing rates).

This motivates a direction-independent measure to quantify the fraction of paths that lead to an increase of correlation with firing rate. This is given exactly in terms of the angle between ∇*Ŝ* and ∇*F*:

14 $$ \cos\theta= \frac{\nabla\hat{S} \cdot\nabla F}{\Vert \nabla \hat{S} \Vert \Vert \nabla F \Vert } $$

and in particular the fraction of **x** directions in which *Ŝ* increases with *F* is given by

15 $$ \frac{1}{\pi} \biggl( \pi-\cos^{-1} \biggl( \frac{\nabla\hat{S} \cdot\nabla F}{\Vert \nabla\hat{S} \Vert \Vert \nabla F \Vert } \biggr) \biggr) . $$

Because cos^−1^ has range $[0, \pi]$, this varies between 0 and 1. The more aligned ∇*Ŝ* and ∇*F*, the more paths lead to $\frac{d\hat{S}}{dF} > 0$; the more anti-aligned ∇*Ŝ* and ∇*F*, the more paths lead to $\frac{d\hat{S}}{dF} < 0$.

### Strength of Recurrent Excitation Modulates the Correlation–Firing Rate Relationship

Our use of inhibitory susceptibility (i.e. Eq. (10)) to characterize the relationship between correlations and firing rates relied on intermediate assumptions, specifically:

- Second-order motifs dominate pairwise correlations.
- Inhibitory common input is the dominant second-order motif.
- Asynchronous spiking assumption: $\operatorname {Var}_{T}(n_{i}) = T \nu_{i} \Rightarrow\tilde{\mathbf {C}}_{ii} = \nu_{i}$.

Here, we check that these conditions are still satisfied for a range of neural network models.

In [4], we examined two spiking regimes achieved by varying the strength of excitation: both recurrent excitation $W_{EE}$ and excitatory input into the inhibitory population $W_{IE}$. We next applied our theory to a dense grid of parameters (different networks), each identified by its location on the $(W_{EE}, W_{IE})$ plane. Both excitatory strengths were varied from a minimum of their values for the asynchronous network ($W_{EE} = 0.5$ and $W_{IE} = 5$) to a maximum of 1.4 times their value in the strong asynchronous network (to $W_{EE} = 12.6$ and $W_{IE} = 11.2$). The parameter $W_{XY}$ is a dimensionless scale factor (see Eqs. (17)–(20)); for example in an all-to-all homogeneous network, $W_{XY}=1$ is when the presynaptic input is exactly the average population firing rate (filtered by the synapse).

To quantify the importance of paths of different length through the network, we can define the contributions at any specific order *k* by using the interaction network **K**:

16 $$\begin{aligned} \tilde{\mathbf {R}}_{ij}^{k} = & \frac{ ( \sum_{l=0}^{k} \tilde{\mathbf {K}}^{l} \tilde{\mathbf {C}}^{0} (\tilde{\mathbf {K}}^{*})^{k-l} )_{ij}}{\sqrt {\tilde{\mathbf {C}}_{ii} \tilde{\mathbf {C}}_{jj} }} . \end{aligned}$$

Then we regressed the total correlation ($\tilde{\mathbf {C}}_{ij}/\sqrt {\tilde{\mathbf {C}}_{ii}\tilde{\mathbf {C}}_{jj}}$) against the contributions at each specific order ($\tilde{\mathbf {R}}^{k}_{ij}$); the corresponding fraction of variance explained ($R^{2}$ value) gives a quantitative measure of how well the total correlation can be predicted from each term.

**Fig. 6:**
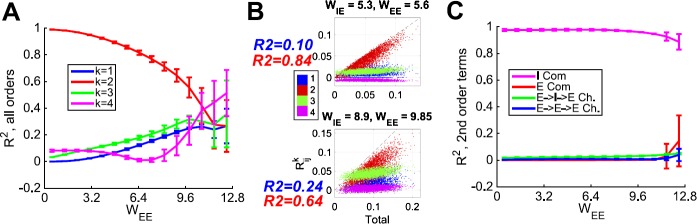
Second-order motifs dominate pairwise correlations in a wide range of networks; inhibitory common input is the dominant second-order motif. (A) Fraction of variance explained ($R^{2}$) from linear regressions of total correlation ($\tilde{\mathbf {C}}_{ij}/\sqrt{\tilde {\mathbf {C}}_{ii}\tilde{\mathbf {C}}_{jj}}$) against contributions from first order (blue), second order (red), third-order (green), and fourth-order (magenta) motifs. (B) Contributions up to fourth order ($\tilde{\mathbf {R}}^{k}_{ij}$, for $k=1,\ldots,4$) vs. total correlation ($\tilde{\mathbf {C}}_{ij}/\sqrt{\tilde {\mathbf {C}}_{ii}\tilde{\mathbf {C}}_{jj}}$) for all E-E cell pairs in a network, for two individual networks included in panel A. (C) Fraction of variance explained ($R^{2}$) from linear regressions of contributions to pairwise correlations from second-order motifs ($\tilde{R}^{2}_{ij}$) against contributions from the distinct types of second-order motifs: inhibitory common input (magenta), excitatory common input (red), decorrelating chains (green), and correlating chains (blue). Both (A,C): Each data point represents the mean value from 15 networks with $W_{IE}$ between 5 and 11.2; error bars show standard deviation across these values. $W_{XY}=1$ is when the presynaptic input is exactly the average population firing rate (filtered by the synapse) in an all-to-all homogeneous network

Similarly, we quantity the importance of specific second-order motif types, by regressing the total contribution from second-order motifs ($\tilde{\mathbf {R}}^{2}_{ij}$) against the contribution from specific motifs. We performed this computation for each network (a total of 225 networks), and summarize the results in Fig. 6; to present the data compactly, we collapse $R^{2}$ values (all values are $\in[0,1]$) for a fixed $W_{EE}$ and varying $W_{IE}$ by showing mean and standard deviation only (standard deviation as error bars). Second-order contributions dominate for small to moderate $W_{EE}$ (Fig. 6(A)); other orders only compete when $W_{EE}$ has already exceeded the level of the strong asynchronous network (where the network is close to the edge of instability, and at the limit of validity for mean-field, asynchronous assumptions).

To illustrate the meaning of this statistic, in Fig. 6(B) we show contributions up to fourth order ($\tilde{\mathbf {R}}^{k}_{ij}$, for $k=1,\ldots,4$) vs. total correlation ($\tilde{\mathbf {C}}_{ij}/\sqrt{\tilde{\mathbf {C}}_{ii}\tilde{\mathbf {C}} _{jj}}$) for all E–E cell pairs in a network, for two individual networks included in the summary panel. Note that the second-order contributions cluster near the unity line in both cases, indicating that second-order contributions are the best predictor of total correlations, consistent with the $R^{2}$ values stated.

Of the second-order motifs, inhibitory common input is the dominant contribution at any value of $W_{EE}$, except perhaps the last, at which point the excitatory population has unrealistically high firing rates (Fig. 6(C)). To summarize, we have confirmed that far from being limited to a few examples, the conditions we identified in [4] as allowing us to focus on susceptibility to inhibition to explain pairwise correlations, appear to hold up for a variety of networks.

We note that our findings about the relative magnitudes of terms of different orders are purely empirical; that is, they are based on concrete numerical observations, rather than a priori estimates. Thus, it should be reassessed if anything about the parameters or network connectivity is changed. In particular, a likely reason for the relative weakness of first-order terms is that in these networks excitation is almost always weaker than inhibition; while first-order terms are always excitatory, second-order terms can involve excitation and/or (comparatively strong) inhibition.

Having confirmed the validity of our approach, we computed the susceptibility with respect to inhibition, for each of the networks examined in the previous section (because of instability, we restricted these computations to excitatory strengths ×1.2 the values used in [4]). Because background noise values varied slightly, we actually examined two network families; one in which we chose *σ* values as in the asynchronous network, another in which we chose *σ* values as in the strong asynchronous network. Also as in [4], we estimated susceptibility using network-averaged values of $g_{E}$, $g_{I}$, $\sigma_{g_{E}}$, and $\sigma_{g_{I}}$.

**Fig. 7:**
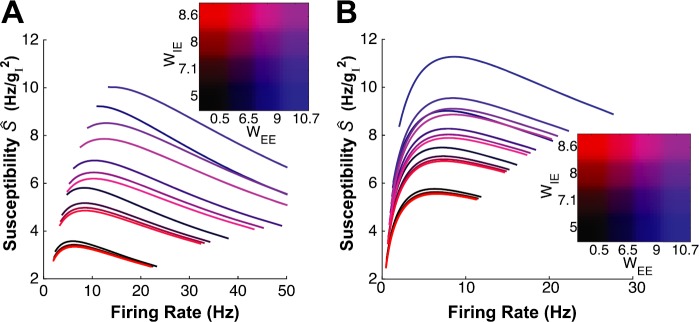
Firing rate vs. susceptibility (*Ŝ*), computed for a family of networks generated by modulating the strength of excitation ($W_{EE}$ and $W_{IE}$). (A) Background noise values $\sigma_{E}$, $\sigma_{I}$ set as in the asynchronous network from [4]. (B) Background noise values $\sigma_{E}$, $\sigma _{I}$ set as in the strong asynchronous network from [4]. Sixteen curves are chosen, for a survey of the range of networks achievable by varying strength of recurrent excitation. Values of $W_{EE}$ are 0.50 (as in the asynchronous network from [4]), 6.45, 9 (as in the strong asynchronous network from [4]), and 10.7. Values of $W_{IE}$ are 5 (as in the asynchronous network from [4]), 7.1, 8 (as in the strong asynchronous network from [4]), and 8.6. Again, $W_{XY}=1$ is when the presynaptic input is exactly the average population firing rate (filtered by the synapse) in an all-to-all homogeneous network, so the coupling strengths vary significantly

Surprisingly, the difference in background noise dwarfed the recurrent excitation strengths, at least in accessing the relationship between *Ŝ* and firing rate. In Fig. 7, we show *Ŝ* vs. *F* curves, for a set of representative networks, on a single plot. Color indicates $W_{EE}$ (shade of blue) and $W_{IE}$ (shade of red); values of $W_{EE}$ are 0.50 (as in the asynchronous network from [4]), 6.45, 9 (as in the strong asynchronous network from [4]), and 10.7, values of $W_{IE}$ are 5 (as in the asynchronous network from [4]), 7.1, 8 (as in the strong asynchronous network from [4]), and 8.6. For reference, $W_{XY}=1$ is when the presynaptic input is exactly the average population firing rate (filtered by the synapse) in an all-to-tall homogeneous network, so these coupling strengths vary significantly. We see that, for the full range of recurrent excitation values, *Ŝ* vs. *F* curves in Fig. 7(A) are mostly decreasing; *Ŝ* vs. *F* curves in Fig. 7(B) are mostly increasing for low *F*, and saturating for high *F*.

### Background Noise Modulates the Correlation–Firing Rate Relationship

**Fig. 8:**
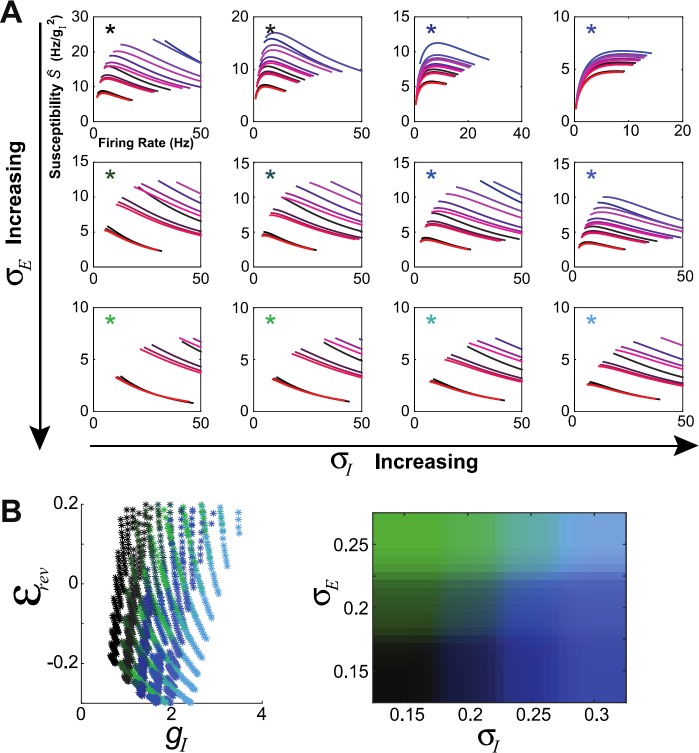
The strength of background noise modulates the correlation–firing rate relationship. (A) Each panel shows firing rate vs. susceptibility (*Ŝ*), computed for a family of networks generated by modulating the strength of excitation ($W_{EE}$ and $W_{IE}$) with various background noise levels (see Eq. (17) for $\sigma_{E}$ and $\sigma_{I}$ definitions). (B) Population-averaged effective parameters $\langle g_{I} \rangle$ and $\mathcal{E}_{\mathrm{rev}}$, for each network displayed in (A); see Eq. (28) for $\mathcal{E}_{\mathrm{rev}}$

To further explore the role of background noise, we repeated the susceptibility calculation on additional families of networks, now allowing background noise strengths $\sigma_{E}$ and $\sigma_{I}$ (i.e. to the excitatory and inhibitory populations) to vary separately. Input to excitatory cells was varied between $\sigma_{E} = 1.5$ and 2.5; input to inhibitory cells was varied between $\sigma_{I} = 1.5$ and 3. These noise values are relatively large; see Eq. (17) and note that voltage is of order 1. In Fig. 8(A) we display susceptibility vs. firing rate curves for 12 $(\sigma_{E}, \sigma_{I})$ pairs; asterisks indicate $\sigma_{E}$ and $\sigma_{I}$ by color (green intensity for $\sigma_{E}$ and blue intensity for $\sigma_{I}$). Within each panel curves are colored as in Fig. 7: red intensity for $W_{IE}$ and blue intensity for $W_{EE}$.

Surprisingly, most network families (i.e. $(\sigma_{E}, \sigma_{I})$) were associated with a *decrease* in correlation with firing rate. The exceptions are $(0.15,0.25)$ (as in the strong asynchronous network in [4]) and $(0.15,0.3)$. This latter was most robustly associated with a positive correlation–firing rate relationship. Furthermore, the shape of susceptibility-firing curves did not appear to vary much with the strength of recurrent excitation (i.e., curves within each panel are similar).

We next sought to investigate possible mechanisms for a positive correlation–firing rate relationship, by examining the effective parameters that govern the neural response: in essence, the network’s “operating point” (see Eq. (26)). Possible choices include $g_{I}$, $g_{E}$, $\sigma_{g_{E}}$, $\sigma_{g_{I}}$, and the effective reversal potential $\mathcal{E}_{\mathrm{rev}}$; we found $\sigma _{g_{E}}$ and $\sigma_{g_{I}}$ to be largely functions of $g_{E}$ and $g_{I}$, while $\mathcal{E}_{\mathrm{rev}}$ has a (nonlinear) functional relationship with $g_{E}$ and $g_{I}$. Thus two parameters suffice, and here we chose to use $g_{I}$ and $\mathcal{E}_{\mathrm{rev}}$. In Fig. 8(B), we plot average parameter values for each curve, color-coded by $(\sigma_{E}, \sigma_{I})$. Any given color is consistent with a relatively tight range of $g_{I}$ and (comparatively) broad range of $E_{\mathrm{rev}}$. As $\sigma_{I}$ increases (increasing blue intensity), inhibitory conductance $g_{I}$ increases and reversal potential $\mathcal{E}_{\mathrm{rev}}$ decreases. However, it was not apparent that any particular region in $(g_{I}, \mathcal{E}_{\mathrm{rev}})$ parameter space was associated with a positive correlation–firing rate relationship, in that the values of $g_{I}$ and $\mathcal{E}_{\mathrm{rev}}$ that supported a positive relationship supported negative relationships as well.

## Discussion

In this paper, we showed that using a single-cell firing rate function to examine the relationship between correlations and firing rates is feasible for a wide range of heterogeneous, recurrent networks. We focused on three factors that can modulate the correlation–firing rate relationship: how the network occupies effective parameter space, strength of recurrent excitation, and strength of background noise. Although there are many sets of parameters known to vary within a heterogeneous network of neurons, we have already demonstrated vastly different correlation–firing rate relationships with our methods, with a theory that can be readily applied to other model networks.

One possible application of this work is in designing neural networks for computational experimentation; just as modelers now modify cortical networks to obey experimental constraints on firing rates [3, 35], we could also include a constraint on the desired correlation–firing rate relationship. Here we showed that we can quickly assess a wide range of possible network configurations for a positive correlation–firing rate relationship: in Sect. 2.5, for example, we performed calculations on $15 \times15 \times12 = 2700$ heterogeneous networks, with a nominal amount of computing time.

One surprising finding in our computations was the relative insensitivity of the slope of the correlation–firing rate relationship to recurrent excitation ($W_{EE}$, $W_{IE}$), as demonstrated in Figs. 7 and 8. This is striking in contrast to the strong sensitivity on display in Figs. 2(B) and 3(B). This difference is explained as follows: in every case where we computed the susceptibility for a self-consistent network (i.e. a solution of Eqs. (26)–(30) and (32)–(33)), the source of heterogeneous firing rates was neural excitability, set via a spiking threshold *θ*. The resulting effective parameters, such as inhibitory conductance $\langle g_{I} \rangle$, did not deviate strongly from their population mean values. In essence, all of these networks took a horizontal path through $(\theta, \langle g_{I} \rangle)$ parameter space, as in Figs. 2(C), (D) and Figs. 3(C), (D). If we were to generate networks where heterogeneity arises from another source—such as the strength or frequency of inhibitory connections [23]—we might see different results. We look forward to exploring this in future work.

A priori, there is no reason to expect that the correlation–firing rate relationship in these recurrent networks can be simplified to a feedforward motif based on shared inhibitory input; this was purely an empirical observation (see Fig. 6(B)). We remark that others have shown that the effective input correlation can be canceled to have near zero input (and thus output) correlation on average in balanced networks [28, 40], in contrast to some of the models considered here (i.e., *strong asynchronous* regime with more net excitation). The conditions for correlation cancellation in these model networks is beyond the scope of this study, but note that others have shown correlation cancellation does not always hold ([21, 22] via altering connection probabilities). The studies that demonstrate correlation cancellation often have faster (or equal) inhibitory synaptic time scales than excitatory: $\tau_{I} \leq\tau_{E}$ [21, 28, 32] ([40] used current-based instantaneous synapses or $\tau_{I}=\tau_{E}=10\mbox{ ms}$) while in our networks the inhibitory synapses have longer time scales (Table 1). Note that Fig. S4 of [28] shows that having effectively zero input correlation does not hold as the inhibitory time scales increase beyond the excitatory time scales. Finally, system size is another key parameter that could certainly affect the magnitude of the recurrent feedback. In contrast to [28, 40], we did not account for how system size would affect correlation cancellation in these heterogeneous networks.

**Table 1:** Intrinsic parameters that are fixed throughout

	Parameter									
	$\tau_{m} $	$\mathcal{E}_{E}$	$\mathcal{E}_{I}$	$\tau _{\mathrm{ref}}$	$\alpha_{E}$	$\alpha_{I}$	$\tau_{r,E}$	$\tau_{d,E}$	$\tau_{r,I}$	$\tau_{d,I}$
Value	20 ms	6.5	−0.5	2 ms	1	2	1 ms	5 ms	2 ms	10 ms

Fixed parameters for model networks; see Eqs. (17)–(20). All are dimensionless except the time scales.

Although affirmative answers to whether correlations increase with firing rate in experiments were cited in the Introduction [2, 5, 8, 11, 19, 36, 44] we also note that many experiments have shown that the average correlation across a population can decrease with firing rate when a global state changes or a stimulus is presented. A recent review [9] shows that stimulus-induced decorrelation (with increased firing rate) occurs in a variety of brain regions and animals. This is slightly different from the situation we examine here, where we consider the relationship between correlations and firing rates within a stimulus condition. Regardless, the fact that the relationship between correlation and firing rate is not obvious points to the continued need for theoretical studies into the mechanisms of spike statistics modulation.

Finally, our finding that correlations often *decrease*, rather than increase, with firing rate stands in apparent contradiction to earlier work on feedforward networks [8, 38]. On closer inspection, we can identify several reasons why our results differ; with conductance inputs (rather than currents) we have a quantitatively different relationship between input parameters and firing rates; furthermore with more adjustable single-neuron parameters, the same sets of firing rates may be observed with single-neuron parameters set in different ways. While the current clamp experiments described in [8] found a consistent increase of correlations with firing rates, we can hypothesize that the parallel dynamic clamp experiments in which pairwise correlations arise from common inhibitory input, would in fact show a decrease with firing rate. However, we also predict that whether an increase or decrease with firing rate is observed would depend on whether firing rates are modulated by varying the level of inhibitory input, or by otherwise varying the excitability of the cells (perhaps pharmacologically).

## Methods

### Neuron Model and Network Setup

We considered randomly connected networks of excitatory and inhibitory neurons. Each cell is a linear integrate-and-fire model with second-order alpha-conductances, i.e. membrane voltage $v_{i}$ was modeled with a stochastic differential equation, as long as the voltage is below threshold $\theta_{i}$:

17 $$\begin{aligned} \tau_{m} \frac{dv_{i}}{dt} =& -v_{i}- g_{E,i}(t) (v_{i}-\mathcal{E}_{E}) - g_{I,i}(t) (v_{i}-\mathcal{E}_{I}) + \sigma_{i} \sqrt{\tau_{m}} \xi_{i}(t). \end{aligned}$$

When $v_{i}$ reaches $\theta_{i}$, a spike is recorded and voltage is reset to 0 following a refractory period:

18 $$\begin{aligned} v_{i}(t) \geq \theta_{i} \quad\Rightarrow\quad v_{i}(t + \tau_{\mathrm{ref}}) = & 0, \end{aligned}$$

Each neuron receives Gaussian white background noise with magnitude $\sigma_{i}$ depending only on the cell type; that is, $\langle\xi_{i}(t) \rangle= 0$ and $\langle\xi_{i}(t) \xi_{i} (t+s) \rangle= \delta(s)$. The membrane time constant, $\tau_{m}$, and excitatory and inhibitory synaptic reversal potentials, $\mathcal{E}_{E}$ and $\mathcal{E}_{I}$, are the same for every cell in the network (see Table 1). The thresholds $\theta_{i}$ are a significant source of heterogeneity, and they are selected from a log–normal distribution with mean 1 and variance $e^{(0.2)^{2}}-1$; since the system size is moderate, the $\theta_{i}$’s were set to have C.D.F. (cumulative distribution function) values equally spaced from 0.05 to 0.95 for both E and I cells.

Each cell responds to synaptic input through conductance terms, $g_{E,i}$ and $g_{I,i}$, which are each governed by a pair of differential equations:

19 $$\begin{aligned} \tau_{d,X} \frac{dg_{X,i}}{dt} = & -g_{X,i} + g^{(1)}_{X,i} , \end{aligned}$$

20 $$\begin{aligned} \tau_{r,X} \frac{dg^{(1)}_{X,i}}{dt} = & -g^{(1)}_{X,i} + \tau _{r,X} \alpha_{X} \biggl( \frac{W_{YX}}{N_{YX}} \biggr)\sum _{j\in X,j \rightarrow i} \sum_{k}\delta(t - t_{j,k}) , \end{aligned}$$

where $Y = \{E,I\}$ denotes the type of cell *i* and $X = \{E,I\}$ denotes the type of the source neuron *j*. Each spike is modeled as a delta-function that impacts the auxiliary variable $g^{(1)}_{X,i}$; here $t_{j,k}$ is the *k*th spike of cell *j*. The rise and decay time constants $\tau_{r,X}$ and $\tau_{d,X}$ and pulse amplitude $\alpha_{X}$ depend only on the type of the source neuron, that is they are otherwise the same across the population. The parameter $W_{YX}$ denotes the strength of $X \rightarrow Y$ synaptic connections, which are (once given the type of source and target neurons) identical across the population. The “raw” synaptic weight (listed in Table 2) is divided by $N_{YX}$, the total number of $X \rightarrow Y$ connections received by each *Y*-type cell.

**Table 2:** Excitatory connection strengths mediate different firing regimes

	Parameter						
	$W_{EI}$ (*I*→*E*)	$W_{IE}$ (*E*→*I*)	$W_{EE}$	$W_{II}$	$\sigma_{E}$	$\sigma_{I} $	Figures
Asynchronous	10	5	0.5	5	$2/\sqrt{2}$	$3/\sqrt{2}$	Figs. 1, 2, 5(A) and (B)
Str. Asynch.	10	8	9	5	$1.5/\sqrt{2}$	$2.5/\sqrt{2}$	Figs. 3, 5(C) and (D)
% connectivity	35 %	20 %	40 %	40 %			

Connectivity details for networks examined in Sect. 2.2. Here $W_{YX}$ denotes *X*→*Y* connections; e.g. $W_{IE}$ denotes the strength of excitatory connections onto inhibitory neurons. The parameter $\sigma_{i}$ denotes the strength of background noise in units of (scaled) voltage, and depends only on cell type (*E* or *I*). The parameters $W_{EE}$, $W_{IE}$, $\sigma_{E}$ and $\sigma_{I}$ will vary in Sects. 2.4 and 2.5; see text for details. For reference, $W_{XY}=1$ is when the presynaptic input is exactly the average population firing rate (filtered by the synapse) in an all-to-tall homogeneous network.

Table 2 show connectivity parameters for the two example networks we discuss in Sect. 2.2. For Figs. 1–3, five parameters are set as stated in this table. In Sect. 2.4 and Figs. 6–7, $W_{EE}$ was varied between 0.5 and 12.6 and $W_{IE}$ between 5 and 11.2. In Sect. 2.5 and Fig. 8, $W_{EE}$ was varied between 0.5 and 10.8 and $W_{IE}$ between 5 and 9.6; $\sigma_{E}$ was varied between 1.5 and 2.5 and $\sigma_{I}$ between 1.5 and 3.

### Linear Response Theory

In general, computing the response of even a single neuron to an input requires solving a complicated, nonlinear stochastic process. However, it often happens that the presence of background noise linearizes the response of the neuron, so that we can describe this response as a perturbation from a background state. This response is furthermore linear in the perturbing input and thus referred to as *linear response* theory [31]. The approach can be generalized to yield the dominant terms in the coupled network response as well. We will use the theory to predict the covariance matrix of spiking activity. The derivation is presented in full in [20, 29, 30]; here, we present only the main points.

We assume we have some way to approximate the change in firing rate which occurs as a result of a change in parameter:

21 $$\begin{aligned} \nu_{i} (t) = & \nu_{i,0} + (A_{X,i} * \epsilon X_{i}) (t); \end{aligned}$$

$\nu_{i,0}$ is the baseline rate (when $X = 0$) and $A_{X,i}(t)$ is a *susceptibility function* that characterizes this firing rate response up to order *ϵ* [8, 20, 41].

In order to consider joint statistics, we need the trial-by-trial response of the cell. First, we propose to approximate the response of each neuron by

22 $$\begin{aligned} y_{i}(t) \approx& y_{i}^{0}(t) + \biggl( A_{X, i} * \sum_{j} (\mathbf {J}_{X,ij} * y_{j} ) \biggr) (t) ; \end{aligned}$$

that is, each input $X_{i}$ has been replaced by a filtered version of the presynaptic firing rates $y_{j}$.

In the frequency domain this becomes

23 $$\begin{aligned} \tilde{y}_{i}(\omega) = & \tilde{y}_{i}^{0} + \tilde{A}_{X, i}(\omega) \biggl( \sum_{j} \tilde{\mathbf {J}}_{X, ij}(\omega) \tilde{y}_{j} (\omega) \biggr), \end{aligned}$$

where $\tilde{y}_{i} = \mathcal{F} [ y_{i} - \nu_{i} ]$ is the Fourier transform of the mean-shifted process ($\nu_{i}$ is the average firing rate of cell *i*) and $\tilde{f} = \mathcal{F} [ f ]$ for all other quantities. In matrix form, this yields a self-consistent equation for *ỹ* in terms of $\tilde{y}^{0}$:

24 $$\begin{aligned} \bigl( \mathbf {I}- \tilde{\mathbf {K}}(\omega) \bigr) \tilde{y} = \tilde {y}^{0} \quad \Rightarrow\quad \tilde{y} = \bigl( \mathbf {I}- \tilde{\mathbf {K}}(\omega) \bigr)^{-1} \tilde{y}^{0}, \end{aligned}$$

where $\tilde{\mathbf {K}}_{ij} (\omega) = \tilde{A}_{X, i}(\omega) \tilde{\mathbf {J}}_{X, ij}(\omega)$ is the interaction matrix in the frequency domain. The cross-spectrum is then computed via

25 $$\begin{aligned} \bigl\langle \tilde{y}(\omega) \tilde{y}^{\ast}(\omega) \bigr\rangle = & \bigl( \mathbf {I}- \tilde{\mathbf {K}}(\omega) \bigr)^{-1} \bigl\langle \tilde {y}^{0}(\omega) \tilde{y}^{0 \ast}(\omega) \bigr\rangle \bigl( \mathbf {I}- \tilde{\mathbf {K}}^{\ast}(\omega) \bigr)^{-1} . \end{aligned}$$

To compute the interaction matrix for a network of conductance-based neurons, we use the effective time constant approximation (as in the supplemental for [41]). We first separate each conductance into mean and fluctuating parts, e.g., $g_{E,i} \rightarrow\langle g_{E,i} \rangle+ ( g_{E,i} - \langle g_{E,i} \rangle )$ (see the discussion in [12]). Next we identify an effective conductance $g_{0,i}$ and potential $\mathcal{E}_{\mathrm{rev},i}$, and treat the fluctuating part of the conductances as noise, i.e. $g_{E,i} - \langle g_{E,i} \rangle \rightarrow\sigma_{g_{E},i} \xi_{E,i}(t)$, so that Eq. (17) becomes

26 $$\begin{aligned} \tau_{m} \frac{dv_{i}}{dt} = & - g_{0,i} (v_{i} - \mathcal{E}_{\mathrm{rev},i}) + \sigma_{g_{E},i} \xi_{E,i}(t) (v_{i} - \mathcal{E}_{E}) \\ &{}+ \sigma_{g_{I},i} \xi_{I,i}(t) (v_{i} - \mathcal{E}_{I}) + \sqrt{\sigma_{i}^{2} \tau_{m}} \xi _{i}(t), \end{aligned}$$

where

27 $$\begin{aligned} g_{0,i} = & 1 + \langle g_{E,i}\rangle+ \langle g_{I,i} \rangle , \end{aligned}$$

28 $$\begin{aligned} \mathcal{E}_{\mathrm{rev},i} = & \frac{ \langle g_{E,i} \rangle\mathcal {E}_{E} + \langle g_{I,i} \rangle\mathcal{E}_{I}}{g_{0,i}} , \end{aligned}$$

29 $$\begin{aligned} \sigma_{g_{E},i}^{2} = & \operatorname {Var}\bigl[ g_{E,i}(t) \bigr] = \mathrm {E}\bigl[ \bigl( g_{E,i}(t) - \langle g_{E,i} \rangle \bigr) ^{2} \bigr] , \end{aligned}$$

30 $$\begin{aligned} \sigma_{g_{I},i}^{2} = & \operatorname {Var}\bigl[ g_{I,i}(t) \bigr] = \mathrm {E}\bigl[ \bigl( g_{I,i}(t) - \langle g_{I,i} \rangle \bigr) ^{2} \bigr] . \end{aligned}$$

The parameters which govern the firing rate response will now be the conductance mean and variance, e.g. $\langle g_{E,i} \rangle$ and $\sigma_{g_{E},i}^{2}$; we next compute how these depend on incoming firing rates for second-order *α*-function synapses (Eqs. (19) and (20)). We first simplify the equation for the auxiliary variable (Eq. (20)):

31 $$\begin{aligned} \tau_{r,X} \frac{dg^{(1)}_{X,i}}{dt} = & -g^{(1)}_{X,i} + \tau _{r,X} \hat{\alpha}_{X,i} \sum _{k} \delta(t-t_{k}) \end{aligned}$$

so that $\hat{\alpha}_{X,i}$ includes all factors that contribute to the pulse size in Eq. (20), including synapse strength and pulse amplitude. The time constants $\tau_{r,X}$, $\tau_{d,X}$ and synapse jump sizes $\hat{\alpha}_{X,i}$ generally depend on cell type. Then assuming that each spike train is a Poisson process with a constant mean firing rate: i.e., each spike train is modeled as a stochastic process $S(t)$ with

$$\bigl\langle S(t) \bigr\rangle = \nu; \qquad \bigl\langle S(t)S(t+\tau) \bigr\rangle - \nu^{2} = \nu\delta(\tau), $$

a straightforward but lengthy calculation shows that

32 $$\begin{aligned} \bigl\langle g_{X,i}(t) \bigr\rangle = & \hat{\alpha}_{X,i} \nu_{X,i} \tau _{r,X} , \end{aligned}$$

33 $$\begin{aligned} \operatorname {Var}\bigl[ g_{X,i}(t) \bigr] = & \biggl( \frac{1}{2} \hat { \alpha}_{X,i}^{2} \nu_{X,i} \tau_{r,X} \biggr) \biggl( \frac{\tau _{r,X}}{\tau_{r,X} + \tau_{d,X}} \biggr) \\ = &\bigl\langle g_{X,i}(t) \bigr\rangle \times\frac{\hat{\alpha}}{2} \times \biggl( \frac{\tau _{r,X}}{\tau_{r,X} + \tau_{d,X}} \biggr) , \end{aligned}$$

where $\nu_{X,i}$ is the total rate of type-*X* spikes incoming to cell *i*. Notice that modulating the rate of an incoming spike train will impact *both* the mean and variance of the input to the effective equation, Eq. (26) (via $\mathcal{E}_{\mathrm{rev},i}$ and $\sigma _{g_{X},i}$). Furthermore, this impact may differ for excitatory and inhibitory neurons, giving us a total of *four* parameters that can be varied in the effective equation.

Therefore, we have four susceptibility functions to compute, $\tilde {A}_{\langle g_{E} \rangle, i}(\omega)$, $\tilde{A}_{\langle g_{I} \rangle, i}(\omega)$, $\tilde{A}_{\sigma_{g_{E}}^{2}, i}(\omega)$, and $\tilde{A}_{\sigma_{g_{I}}^{2}, i}(\omega)$. The first two capture the change in firing rate as a result of a change in mean conductance—$\langle g_{E,i} \rangle\rightarrow\langle g_{E,i} \rangle_{0} + \langle g_{E,i} \rangle_{1} \exp(\imath\omega t)$ or $\langle g_{I,i} \rangle\rightarrow\langle g_{I,i} \rangle_{0} + \langle g_{I,i} \rangle_{1} \exp(\imath\omega t)$—while the final two address a change in variance—$\sigma_{g_{E},i}^{2} \rightarrow ( \sigma_{g_{E},i}^{2} )_{0} + ( \sigma_{g_{E},i}^{2} )_{1} \exp(\imath\omega t)$ or $\sigma_{g_{I},i}^{2} \rightarrow ( \sigma_{g_{I},i}^{2} )_{0} + [4] ( \sigma_{g_{I},i}^{2} )_{1} \exp(\imath\omega t)$. Since the corresponding Fokker–Planck equation required to obtained these entities is linear, we can compute both susceptibilities separately and combine them to get the net effect. With these pieces, we now have the interaction matrix:

34 $$\begin{aligned} \tilde{\mathbf {K}}_{ij} (\omega) = & \textstyle\begin{cases} \tilde{A}_{\langle g_{E} \rangle, i}(\omega) \tilde{\mathbf {J}}_{ij}(\omega) + \tilde{A}_{\sigma_{g_{E}}^{2}, i}(\omega) \tilde {\mathbf {L}}_{ij}(\omega), & j \text{ excitatory,} \\ \tilde{A}_{\langle g_{I} \rangle, i}(\omega) \tilde{\mathbf {J}}_{ij}(\omega) + \tilde{A}_{\sigma_{g_{I}}^{2}, i}(\omega) \tilde {\mathbf {L}}_{ij}(\omega), & j \text{ inhibitory,} \end{cases}\displaystyle \end{aligned}$$

where $\tilde{\mathbf {L}}(\omega)$ plays a similar role as $\tilde{\mathbf {J}}$, but for the effect of incoming spikes on the *variance* of conductance. Its relationship to $\tilde{\mathbf {J}}$ (either in the frequency or time domain) is given by the same simple scaling shown in Eq. (33): i.e., for *j* excitatory,

35 $$\begin{aligned} \tilde{\mathbf {L}}_{ij} (\omega) = & \tilde{\mathbf {J}}_{ij}(\omega) \times \biggl( \frac{\hat{\alpha}_{E,i}}{2} \biggr) \times \biggl( \frac{\tau_{r,E}}{\tau_{r,E} + \tau_{d,E}} \biggr), \end{aligned}$$

where the first factor comes from the effective spike amplitude $\hat {\alpha}_{E,i}$ (and is the scale factor proposed in [29], Eq. (64)), and the second arises from using second-order (vs. first-order) alpha-functions.

To implement this calculation, we first solve for a self-consistent set of firing rates: that is, $\nu_{i}$ is the average firing rate of Eq. (26), along with Eqs. (27)–(30) and (32)–(33). We then apply Richardson’s threshold integration method [29, 30] directly to Eq. (26) to compute the unperturbed power spectrum ($\langle\tilde{y}^{0}(\omega ) \tilde{y}^{0 \ast}(\omega) \rangle$) and susceptibility functions. The software we used to implement this calculation is described more fully in [4] and can be found at https://github.com/andreakbarreiro/LR_CondBased.

### Computing Statistics from Linear Response Theory

Linear response theory yields the cross-spectrum of the spike train, $\langle\tilde{y}_{i}(\omega) \tilde{y}_{j}^{\ast}(\omega) \rangle$, for each distinct pair of neurons *i* and *j* (see Eq. (25)). The cross-correlation function, $\mathbf {C}_{ij}(\tau)$, measures the similarity between two processes at time lag *τ*, while the cross-spectrum measures the similarity between two processes at frequency *ω*:

36 $$\begin{aligned} \mathbf {C}_{ij} (\tau) \equiv& \bigl\langle \bigl(y_{i}(t)- \nu_{i} \bigr) \bigl(y_{j}(t + \tau )-\nu_{j} \bigr) \bigr\rangle , \end{aligned}$$

37 $$\begin{aligned} \tilde{\mathbf {C}}_{ij}(\omega) \equiv& \bigl\langle \tilde{y}_{i} (\omega) \tilde{y}_{j} (\omega) \bigr\rangle . \end{aligned}$$

The Weiner–Khinchin theorem [31] implies that $\{ \mathbf {C}_{ij}, \tilde{\mathbf {C}}_{ij} \} $ are a Fourier transform pair: that is,

38 $$\begin{aligned} \tilde{\mathbf {C}}_{ij} (\omega) = & \int_{-\infty}^{\infty} \mathbf {C}_{ij} (t) e^{-2 \pi\imath\omega t}\, dt . \end{aligned}$$

In principle, the cross-correlation $\mathbf {C}(t)$ and cross-spectrum $\tilde{\mathbf {C}}(\omega)$ matrices are functions on the real line, reflecting the fact that correlation can be measured at different time scales. In particular, for a stationary point process the covariance of spike counts over a window of length *T*, $n_{i}$ and $n_{j}$, can be related to the cross-correlation function $\mathbf {C}_{ij}$ by the following formula [17]:

39 $$\begin{aligned} \operatorname {Cov}_{T}(n_{i}, n_{j}) = & \int_{-T}^{T} \mathbf {C}_{ij}(\tau) (T - \vert \tau\vert )\, d\tau . \end{aligned}$$

The variance of spike counts over a time window of length *T*, $n_{i}$, is likewise given by integrating the autocorrelation function $\mathbf {C}_{ii}$:

40 $$\begin{aligned} \operatorname {Var}_{T}(n_{i}) = & \int_{-T}^{T} \mathbf {C}_{ii}(\tau) (T - \vert \tau\vert )\, d\tau . \end{aligned}$$

By normalizing by the time window and taking the limit as $T\rightarrow \infty$,

41 $$\begin{aligned} \lim_{T \rightarrow\infty} \frac{\operatorname {Cov}_{T}(n_{i}, n_{j})}{T} = & \lim_{T \rightarrow\infty} \int_{-T}^{T} \mathbf {C}_{ij}(\tau) \biggl(1 - \frac{\vert \tau\vert }{T} \biggr)\, d\tau \\ = & \int_{-\infty}^{\infty} \mathbf {C}_{ij}(\tau)\, d\tau = \tilde{\mathbf {C}}_{ij}(0) , \end{aligned}$$

we can see that, for an integrable cross-correlation function, we can use $\tilde{\mathbf {C}}_{ij}(0)$ as a measure of long-time covariance.

Similarly, the long-time limit of the Pearson correlation coefficient of the spike counts,

42 $$\begin{aligned} \lim_{T \rightarrow\infty} \rho_{T,ij} = & \lim_{T \rightarrow \infty} \frac{\operatorname {Cov}_{T}(n_{i},n_{j})}{\sqrt{ \operatorname {Var}_{T}(n_{i}) \operatorname {Var}_{T}(n_{j})}} = \frac{\tilde{\mathbf {C}}_{ij}(0)}{\sqrt{\tilde{\mathbf {C}}_{ii}(0) \tilde {\mathbf {C}}_{jj}(0)}} , \end{aligned}$$

gives us a normalized measure of long-time correlation.

## Abbreviations

E: excitatory
I: inhibitory
Asyn: asynchronous
Strong Asyn: strong asynchronous
